# In-Hospital and Long-Term Prognosis after Myocardial Infarction in Patients with Prior Coronary Artery Bypass Surgery; 19-Year Experience

**DOI:** 10.1100/tsw.2009.114

**Published:** 2009-10-01

**Authors:** Predrag M. Mitrovic, Branislav Stefanovic, Zorana Vasiljevic, Mina Radovanovic, Nebojsa Radovanovic, Gordana Krljanac, Ana Novakovic, Miodrag Ostojic

**Affiliations:** Division of Emergency Cardiology of the University Institute for Cardiovascular Diseases, Clinical Center of Serbia, School of Medicine, University of Belgrade, 11000 Belgrade, 8 Koste Todorovica, Serbia

**Keywords:** acute myocardial infarction, revascularization, prognosis

## Abstract

To present a 19-year experience of the prognosis of patients with acute myocardial infarction (AMI) and prior coronary artery bypass surgery (CABS), 748 patients with AMI after prior CABS (postbypass group) and a control group of 1080 patients with AMI, but without prior CABS, were analyzed. All indexes of infarct size were lower in the postbypass group. There was more ventricular fibrillation in the postbypass group. In-hospital mortality was similar (*p* = 0.3675). In the follow-up period, postbypass patients had more heart failure, recurrent CABS, reinfarction, and unstable angina than did control patients. Cumulative survival was better in the control group than in the postbypass group (*p* = 0.0403). Multiple logistic regression model showed that previous angina (*p* = 0.0005), diabetes (*p* = 0.0058), and age (*p* = 0.0102) were independent predictor factors for survival. Use of digitalis and diuretics, together with previous angina, also influenced survival (*p* = 0.0092), as well as male gender, older patients, and diabetes together (*p* = 0.0420). Patients with AMI after prior CABS had smaller infarct, but more reinfarction, reoperation, heart failure, and angina. Previous angina, diabetes, and age, independently, as well as use of digitalis and diuretics together with angina, and male gender, older patients, and diabetes together, influenced a worse survival rate in these patients.

## INTRODUCTION

The long-term fate of bypass vein grafts is principally determined by graft atherosclerosis. Atherosclerosis frequently develops in native vessel[1,2,3,4] and in vein grafts[5.6] after coronary artery bypass surgery (CABS). This process appears to be related to age, gender, or coronary risk factors. CABS may result in reperfusion injury, causing cardiac failure. Delay, however, may risk a second ischemic event. Timing of acute myocardial infarction (AMI) after CABS is controversial, especially if myocardial function is depressed after CABS. However, little information is available on the in-hospital and long-term effect of bypass surgery on the incidence of myocardial infarction.

The aim of this study was to present a 19-year experience of the early and late prognosis of patients with AMI and prior CABS.

## METHODS

Data from all patients (n = 1828) diagnosed with AMI at the University Institute for Cardiovascular Diseases, Clinical Center of Serbia between 1988 and 2007 were prospectively collected and entered into a database. Seven hundred and forty-eight patients (40.9%) were admitted to the coronary care unit 98.2 ± 16.2 months after CABS with diagnosis of AMI. The diagnostic criteria and coronary care unit protocol were identical for all patients. For inclusion, patients were required to have chest pain with verification by diagnostic electrocardiographic (ECG) changes (new ST-elevation >0.2 mV in two contiguous leads and >0.1 mV in two contiguous other leads, ST in leads depression >0.1 mV in two contiguous leads in the absence of QRS confounders, new left bundle branch block, or dynamic T-wave inversion in more than one lead), cardiac enzyme changes (troponin T measurement >0.01 µg/l or a creatine kinase measurement more than twice the upper range of normal for the measuring laboratory)[7].

The patients were divided into two groups: a postbypass group, 748/1828 (40.9%) with AMI after previous CABS; a control group, 1080/1828 (59.1%) with AMI without previous CABS. The patients with early perioperative AMI (within 30 days after operation) were excluded from the study. This AMI was treated as a complication of the cardiac surgery.

Demographic, historical, and clinical data were recorded during hospitalization on designated forms. Infarct size was estimated by (1) peak of cardiospecific enzyme, (2) ECG ST segment elevation (both the maximal number of leads showing ST elevation at 60 msec after the J point and the sum of ST elevation in the 12 leads), and (3) the modified QRS score of Wagner et al.[8] obtained from the 12-lead ECG between days 7 and 10. Q wave and ST segment abnormalities persisting from a previous AMI were not counted.

After AMI, all patients returned to our institution for follow-up 1 month after discharge, after an additional 2 months, and every 6 months thereafter. The control protocol was identical in both groups. The mean duration of follow-up in both groups was 114 ± 21 months (range: 6–180). End point was patient death.

### Statistical Analysis

Continuous data are expressed as median values and categorical data are presented as a percent. Categorical variables were compared using the Chi-square test or Fisher exact test. Continuous variables were compared using Student's *t*-test. $P$ value < 0.05 was considered as significant.

Patients with previous CABS vs. patients without previous CABS were compared and variables with some predictive value were then tested in a multiple logistic regression model to identify independent and nonindependent predictors of long-term mortality.

## RESULTS

In the study period (1988–2007), of 1828 consecutive AMI patients, a total of 748 (40.9%) patients had previous CABS (postbypass group). Out of the remaining AMI patients without previous CABS, 1080 patients, consecutively admitted, were included in the study as the control group.

## Baseline Characteristics

The baseline data are shown in Table 1. The mean interval between CABS and AMI in the 748 postbypass patients was 98.2 months (range: 2–240) with 3.2 grafts/patient. There were more men in the postbypass group (87 vs. 79%, $p$ = 0.0001). The postbypass patients had a statistically significant greater prevalence of smoking, family history, total cholesterol, body mass index, as well as previous infarction, post-CABS angina, and the use of drugs that might influence infarct size, except ACE inhibitors. The use of beta-blockers, digitalis, and nitrates was statistically significant more often in postbypass patients. Control patients used diuretics, Ca-antagonists, anticoagulants, and aspirin more often, which was statistically significant. ACE inhibitors were used more often in the postbypass group, but there was no statistically significant difference between the two groups ($p$ = 0.2002). The age, prevalence of hypertension, diabetes mellitus, HDL cholesterol, triglycerides, and previous congestive heart failure were similar in the two groups.

**Table 1.** Comparison of baseline data in the two study groups

| | Postbypass (n = 748) | Control (n = 1080) | p Value |
|---|---|---|---|
| Age (years) | 59.6±5.8 | 61.2±5.2 | 0.5248 |
| Male/Female | 653/95 | 853/227 | 0.0001 |
| Coronary risk factors | | | |
| Hypertension | 33.9% | 35% | 0.6449 |
| Smoking | 14.9% | 22% | 0.0002 |
| Diabetes mellitus | 26.6% | 28% | 0.5220 |
| Family history | 39.7% | 27.8% | 0.0001 |
| Total cholesterol (mmol/liter) | 6.6 ± 1.4 | 7.2 ± 1.0 | 0.0015 |
| HDL cholesterol (mmol/liter) | 1.1 ± 0.6 | 1.3 ± 0.4 | 0.4800 |
| Triglycerides (mmol/liter) | 2.4 ± 1.6 | 2.8 ± 1.0 | 0.3200 |
| Body mass index (kg/m$^2$) | 25.2 ± 2.2 | 27 ± 2.8 | 0.0090 |
| Previous infarction | 45.9% | 34% | 0.0001 |
| Post CABS angina pectoris | 42.9% | 38% | 0.0336 |
| Previous congestive heart failure | 18.8% | 22.5% | 0.0596 |
| Preadmission medications | | | |
| Beta-blocking agents | 82% | 64.2% | 0.0001 |
| Digitalis | 51.9% | 29.6% | 0.0001 |
| Diuretics | 22.1% | 32% | 0.0001 |
| Nitrates | 82% | 78% | 0.0375 |
| Calcium channel blocking agents | 17.9% | 33% | 0.0001 |
| ACE inhibitors | 23.9% | 21.4% | 0.2002 |
| Aspirin | 46% | 51.2% | 0.0283 |
| Anticoagulants | 11.9% | 37% | 0.0001 |

## In-Hospital Characteristics

The infarct size and in-hospital complications in the two study groups of the patients are shown in Table 2. All enzymatic and ECG indexes of infarct size were lower in the postbypass group (all *p* values = 0.0001), except QRS score (similar in both groups). Non-Q wave infarction was more common in the postbypass patients than in the control patients (64 vs. 32.8%, $p = 0.0001$), as well as inferior AMI (53 vs. 44%, $p = 0.0096$).

**Table 2.** Comparison of infarct size and in-hospital complications in the two study groups

| | Postbypass (n = 748) | Control (n = 1080) | p Value |
|---|---|---|---|
| Infarct size assessment (mean±SD) | | | |
| Maximal CK (IU/liter) | 1.110±896 | 1.680±1.410 | 0.0001 |
| ECG leads with ST elevation | 1.52±1.8 | 2.5±2.0 | 0.0001 |
| Maximal summed ST elevation (mm) | 1.52±1.8 | 2.5±2.0 | 0.0001 |
| Leads with new Q waves | 0.96±1.48 | 2.0±1.8 | 0.0001 |
| QRS score | 1.65±2.60 | 1.62±2.70 | 0.6800 |
| Infarct location | | | |
| Anterior (A) | 42% | 46% | 0.2442 |
| Inferior (I) | 53% | 44% | 0.0096 |
| Combined (A+I) | 5% | 10% | 0.0108 |
| Non-Q wave infarction | 64% | 32.8% | 0.0001 |
| Ejection fraction (EF) | 46±10% | 58±8% | 0.0332 |
| Coronarography findings | | | |
| 1-vessel disease | 8.3% | 26.5% | 0.0001 |
| 2-vessel disease | 74.3% | 39.8% | 0.0001 |
| 3-vessel disease | 17.4% | 33.7% | 0.0001 |
| In-hospital complications | | | |
| Ventricular fibrillation | 8.9% | 5.8% | 0.0106 |
| Severe congestive heart failure | 6% | 7% | 0.3880 |
| $2^{nd}$ and $3^{rd}$ degree AV block | 2.9% | 5.8% | 0.0039 |
| Death | 4.9% | 5.9% | 0.3675 |

Ejection fraction was significantly lower statistically in the postbypass group than in the control group (46 vs. 58%, $p = 0.0332$).

Coronary angiography showed that the postbypass group had two-vessel (74.3 vs. 39.8%, $p = 0.0001$) disease more often, which was statistically significant, contrary to the control group that had one-vessel (8.3 vs. 26.5%, $p = 0.0001$) and three-vessel disease (17.4 vs. 33.7%, $p = 0.0001$) more often. These findings presented that the postbypass group of patients had statistically significant more frequent multivessel (two- or three-vessel) disease than the control group of patients (91.7 vs. 73.5%, $p = 0.0001$).

The frequency of ventricular fibrillation was significantly different between the postbypass and control patients (8.9 vs. 5.8%, $p = 0.0106$), as well as second- and third-degree AV block (2.9 vs. 5.8%, $p = 0.0039$). The frequency of severe congestive heart failure and death was not significantly different in the two groups.

## Clinical Outcome

Overall, 37 (28%) postbypass and 64 (23.6%) control patients died during a mean follow-up period of 108 ± 14 months ($p = 0.0403$) (Table 3). In both groups, about 82% of the deaths were of cardiac origin. However, the mechanism of death was much more likely to be sudden in postbypass than in control patients (56.3 vs. 20.7%, $p = 0.0001$). The Kaplan-Meier probability of survival at 19 years after infarction was 72% in the postbypass group and 76.4% in the control group, respectively (Fig. 1). There was significant difference in survival between these two groups ($p = 0.0403$).

**Table 3.** Cause of death in the postbypass and control groups with AMI

| | Postbypass (n = 711) | Control (n = 1016) | p Value |
|---|---|---|---|
| Total deaths | 28% | 23.6% | 0.0403 |
| Cardiac | 83.9% | 80.4% | 0.3416 |
| Sudden | 56.3% | 20.7% | 0.0001 |
| Non cardiac | 14% | 15.4% | 0.6926 |
| Unknown | 2.1% | 4.2% | 0.2005 |

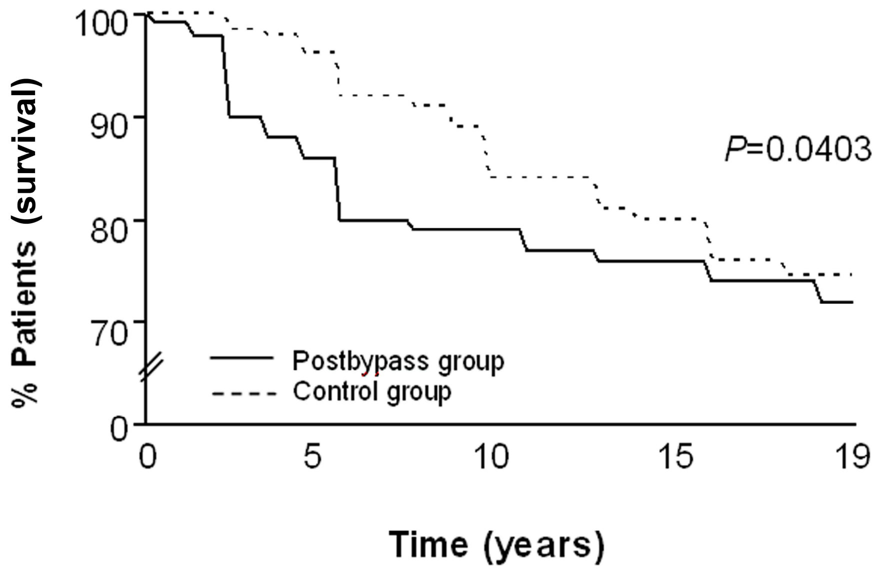

**FIGURE 1.** Cumulative percent survival after AMI for the postbypass surgery and control groups.

During the follow-up period, the postbypass patients experienced more reinfarctions than did the control patients (39.9 vs. 18.6%, $p = 0.0001$) (Table 4). Unstable angina also occurred more often in the postbypass patients (24.8 vs. 18.6%, $p = 0.0014$). Coronary revascularization, usually bypass surgery, was performed within 19 years in 25.8% of the postbypass patients compared with 16.2% of the control patients ($p = 0.0001$). Overall, total cardiac events were significantly more common in the postbypass group ($p = 0.0001$).

**Table 4.** Clinical outcome at 19-year follow-up period

| | Postbypass (n = 711) | Control (n = 1016) | p Value |
|---|---|---|---|
| Reinfarction | 39.9% | 18.6% | 0.0001 |
| Unstable angina | 24.8% | 18.6% | 0.0014 |
| Heart failure | 22.6% | 17.8% | 0.0159 |
| Revascularization | 25.8% | 16.2% | 0.0001 |
| Cardiac mortality | 83.9% | 80.4% | 0.3416 |
| All events | 70.2% | 50.4% | 0.0001 |

Multiple logistic regression model showed that previous angina ($p = 0.0005$), diabetes ($p = 0.0058$), and age ($p = 0.0102$) were independent predictor factors for survival. Use of digitalis and diuretics, together with previous angina, also influenced survival ($p = 0.0092$), as did male gender, older patients, and diabetes together ($p = 0.0420$).

## DISCUSSION

Graft atherosclerosis accelerates several years after coronary artery bypass grafting. The development and progression of atherosclerosis in the saphenous vein grafts[2] led to both a relative and absolute increase in the number of patients having AMI after CABS. The annual incidence of AMI after CABS ranges from 3 to 8%, according to the findings of various studies[4,9,10,11,12].

Crean et al.[13] compared infarct size and determined the cause of infarction in 52 control patients and in 52 consecutive patients who had an AMI at least 2 months after CABS. Baseline characteristics were similar in both groups except for a higher incidence of pre-existing Q waves in the post-CABS group. Indexes of the AMI size were smaller, as in our study, and the postinfarction left ventricular ejection fraction was higher in patients with previous CABS than in controls. In our study, postinfarction ejection fraction was significantly higher statistically in the control group of patients ($p = 0.0332$), probably because of more frequent expression of multivessel disease in the postbypass group of patients ($p = 0.0001$).

Grines et al.[10] retrospectively reviewed the medical records of 50 patients previously treated by CABS, who had cardiac catheterization or autopsy within 1 week of a subsequent AMI. Of these patients, 47 (84%) were men. Cardiac risk factors at the time of AMI included cigarette smoking (83%), systemic hypertension (66%), and diabetes mellitus (17%). Similar to our study, there was a history of previous AMI in 25 (50%), and angina occurring within the previous week was present in 23 patients (46%). Four patients died during hospitalization for AMI.

In their prospective study, Davis et al.[14] followed the incidence of AMI in medically and surgically treated patients from the National Heart, Lung, and Blood Institute Coronary Artery Surgery Study (CASS) Registry. There were 985 AMI patients who had not had CABS and 369 AMI patients who had. The 30-day mortality from AMI was 21% in the surgical group and 36% in the medical group. Death occurred within 24 h after AMI in 81% of the surgical group and 80% of the medical group; 94% of both groups died within 1 week of AMI.

In our study, the postbypass patients had a statistically significant prevalence of use of drugs that might influence infarct size, except ACE inhibitors. ACE inhibitors were also used more often in the postbypass group of patients, but there was no statistically significant difference between the two groups ($p = 0.2002$).

On comparing 219 patients who had AMI and a history of CABS with 2275 patients who had AMI without previous surgery, as in our study, Dittrich et al.[15] showed that the first group had a statistically significant increase in prevalence of a history of previous AMI (70 vs. 24%), congestive heart failure (22 vs. 10%), and angina pectoris (75 vs. 35%). Patients with previous AMI were predominantly men, 88 vs. 75%; there was no difference between the groups in terms of age. During hospitalization, as in our study, surgical patients had more recent angina pain (46 vs. 32%, $p < 0.001$) and more frequently had non-Q wave AMI (33 vs. 23%, $p < 0.01$). In-hospital mortality did not differ among the two groups of patients: 7% in the surgically treated patients and 9% in patients without previous CABS. At discharge, a significantly larger number of patients with previous CABS had complex ventricular arrhythmias and depressed left ventricular ejection fraction (<45%), as in our study. One-year cardiac mortality after AMI was significantly higher in patients who had had CABS, 16 vs. 8% ($p < 0.01$).

Long-term prognosis after AMI in patients with previous CABS was evaluated in a group of 205 patients hospitalized with AMI 2–162 months after CABS and in 205 controls[16]. At baseline, the post-CABS group consisted of more men and more patients with a previous AMI. Indexes of infarct size were lower in patients who had had CABS; however, post-AMI left ventricular ejection fraction, extent of coronary artery disease, and in-hospital mortality rate were similar in both groups. The patients had follow-up for 34 ± 25 months after hospital discharge; at 5 years after dismissal, cumulative mortality was similar in both groups, but as in our study, postbypass patients had more reinfarction, more rehospitalization for unstable angina, and more revascularization procedures than did the control patients.

The 10-year incidence of AMI and the prognosis after infarction were evaluated in 686 patients with stable angina who were randomly assigned to medical or surgical treatment in the Veterans Administration Cooperative Study of Coronary Artery Bypass Surgery[17]. During the follow-up period, immediate risk of death after AMI was 59% lower in the surgical than in the medical patients, and the surgical group had a considerably better cardiac risk profile; however, the cumulative mortality or myocardial infarction incidence was higher in the surgical group. The reduction of postinfarction mortality after CABS was more striking in the first month after AMI (99%) and dropped considerably (48%) afterward. The authors emphasized that although CABS surgery does not reduce the overall incidence of AMI, it does reduce the risk of mortality after infarction, particularly in the first 30 days after the event.

In our study, despite more Q wave infarcts, only 16.2% of the control patients underwent revascularization, probably because of the less-severe nature of coronary disease. The control patients had statistically significant more frequent one-vessel disease than the postbypass patients. The postbypass patients had statistically significant more frequent multivessel disease.

## CONCLUSIONS

Despite the large number of separate studies published on CABS and AMI, there are few randomized studies of AMI in patients who have had CABS. Myocardial infarction after CABS occurs more frequently in older patients, and most of them are men. Moreover, previous AMI, heart failure, and angina pectoris are more frequent. When patients with CABS have an AMI, the infarct size, compared with that in patients without previous CABS, is smaller. On 19-year follow-up, more postbypass patients had AMI, anginal syndrome, heart failure, and revascularization procedures. The survival 19 years after infarction was better in patients with AMI and without previous CABS. Previous angina, diabetes, and age, independently, as well as use of digitalis and diuretics together with angina, and male gender, older patients, and diabetes together, influenced a worse survival rate in patients with AMI and previous CABS.
